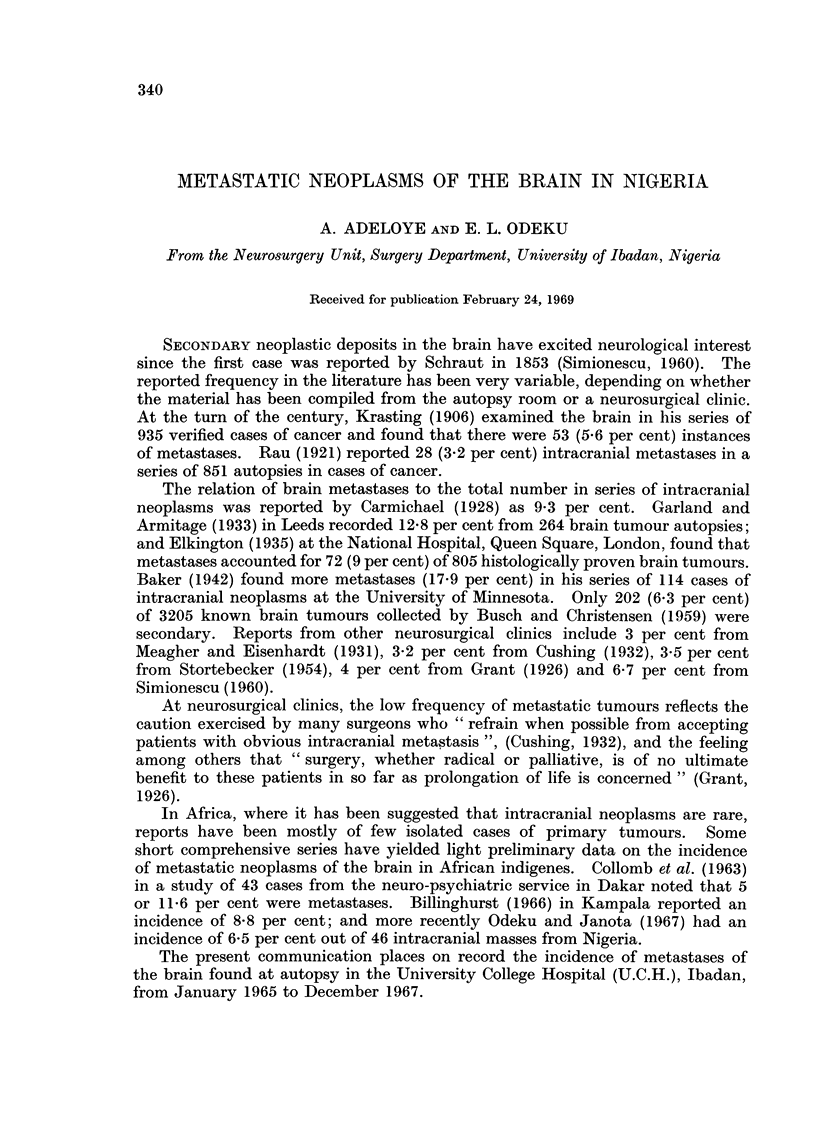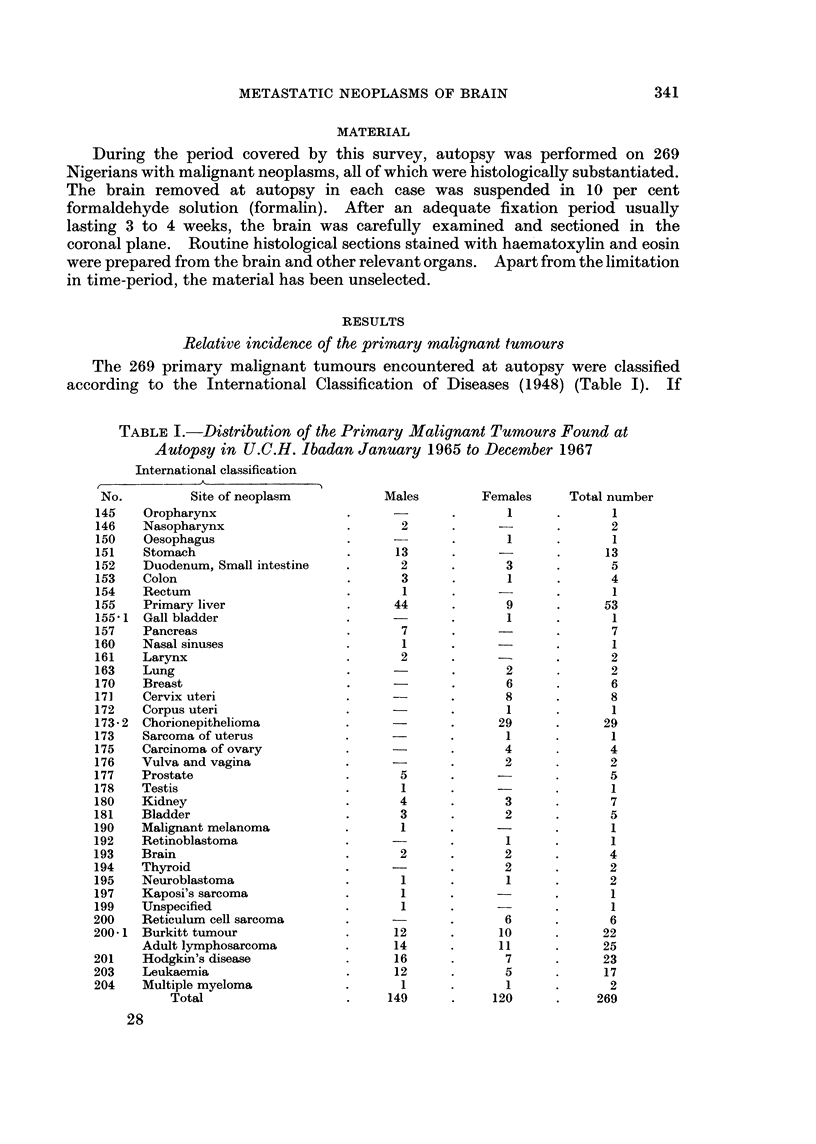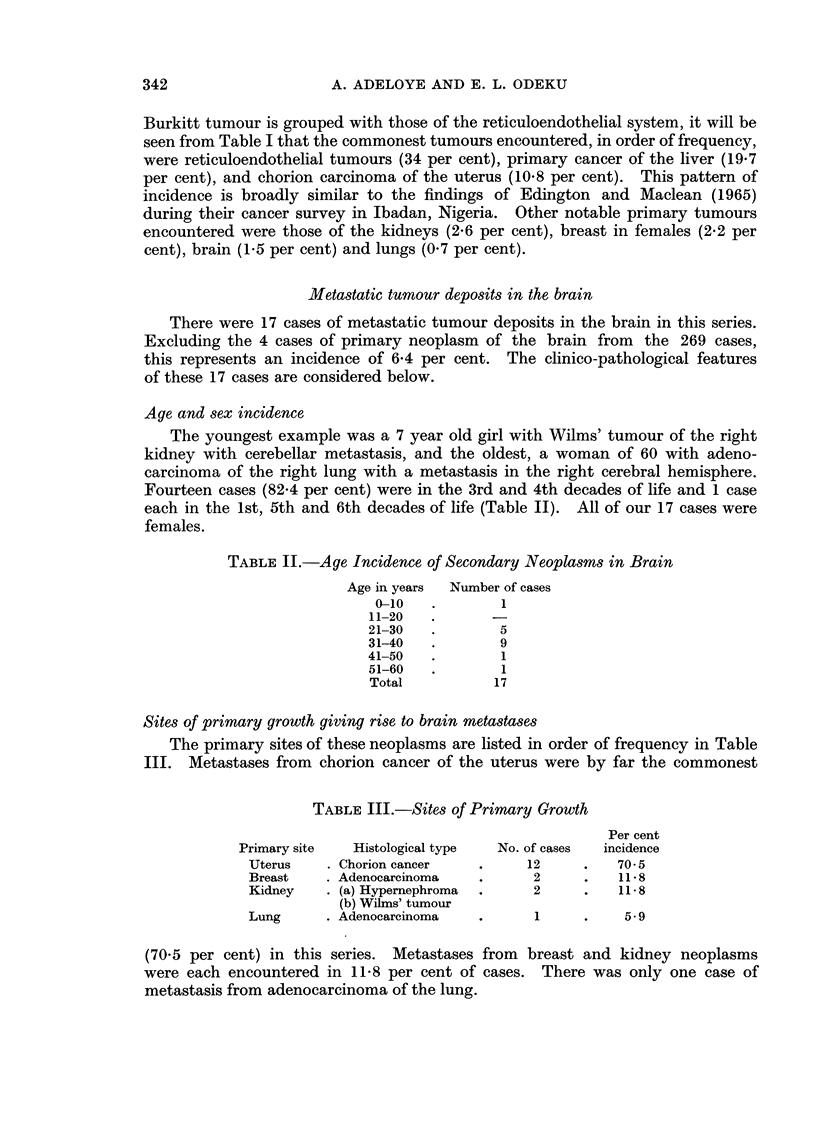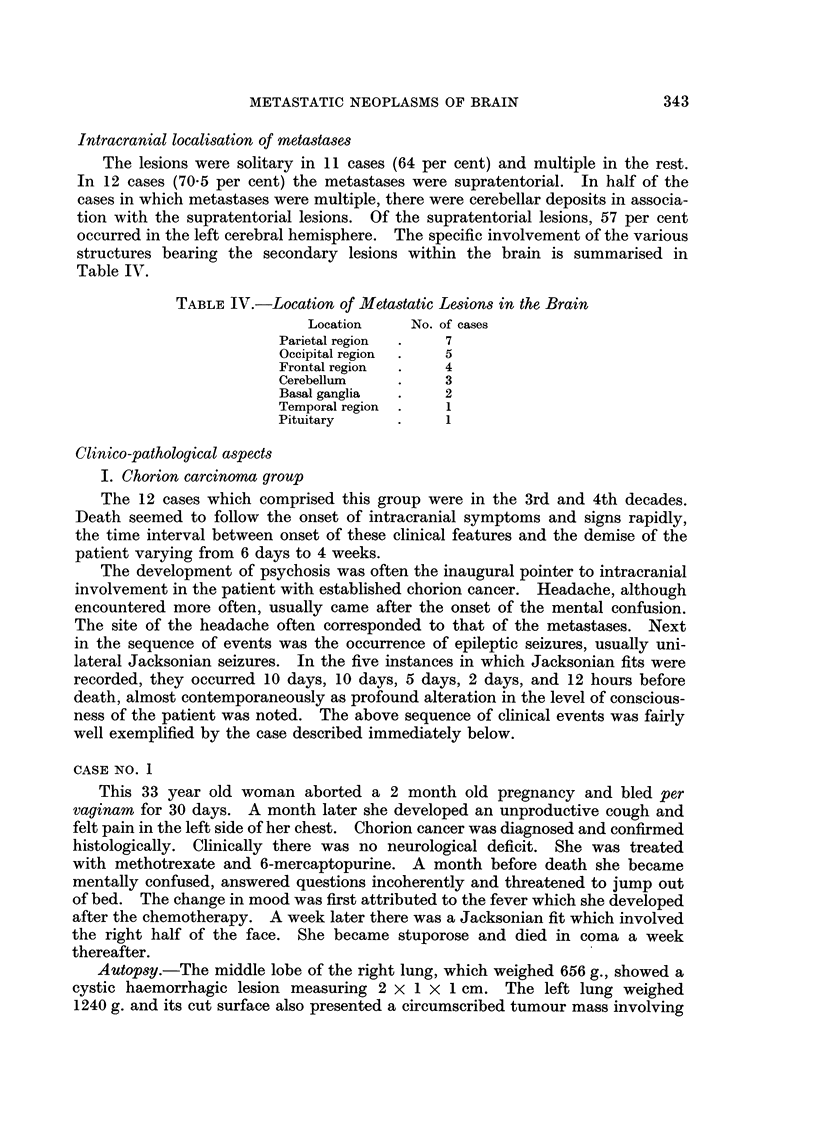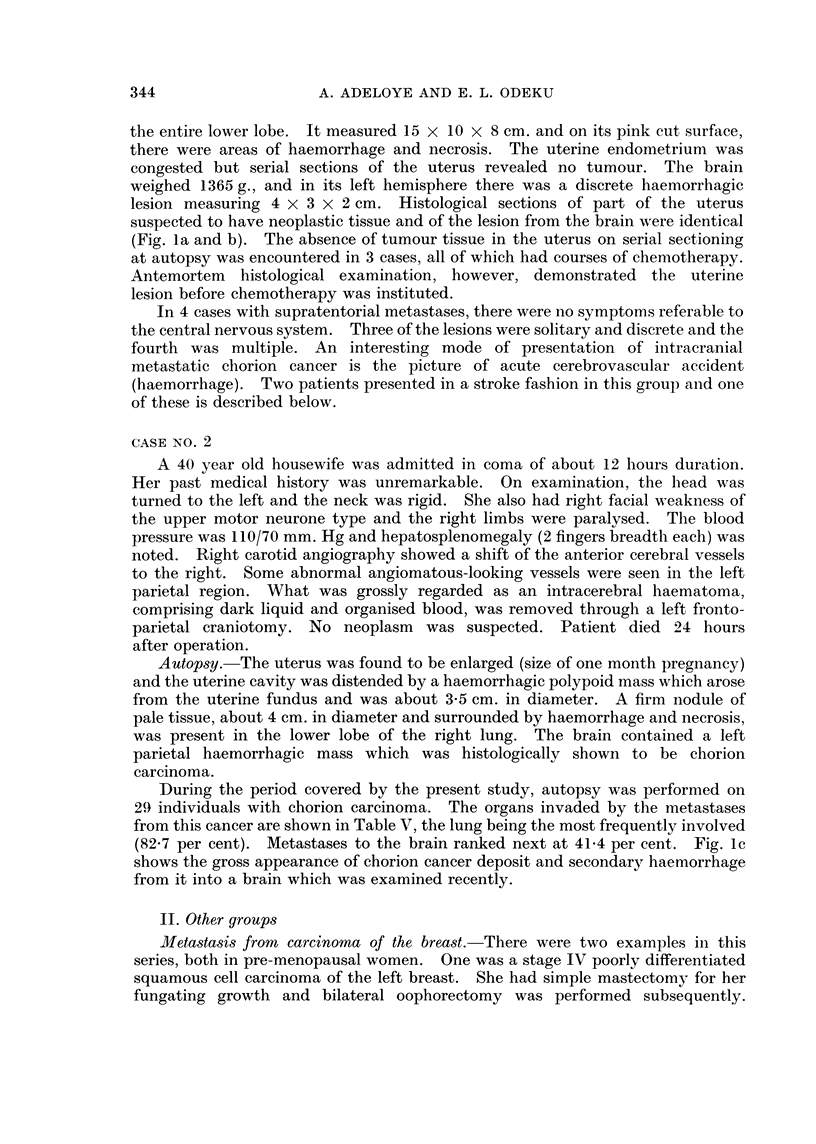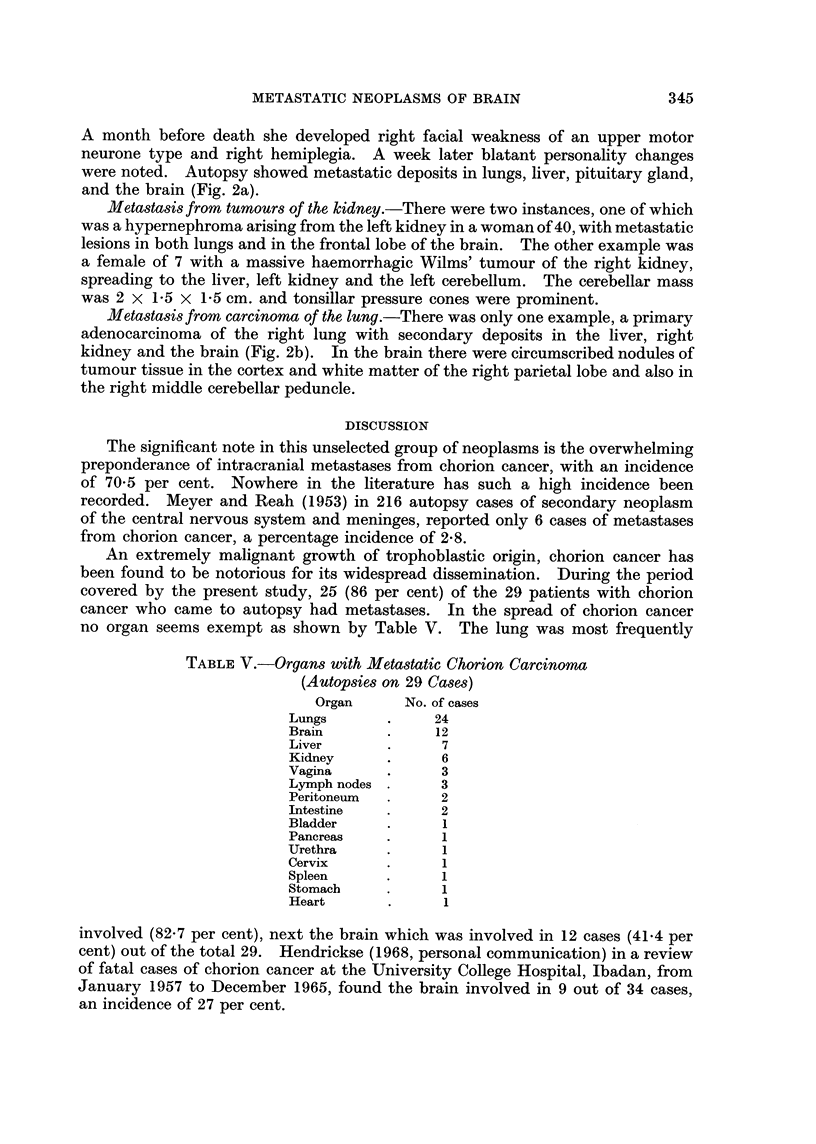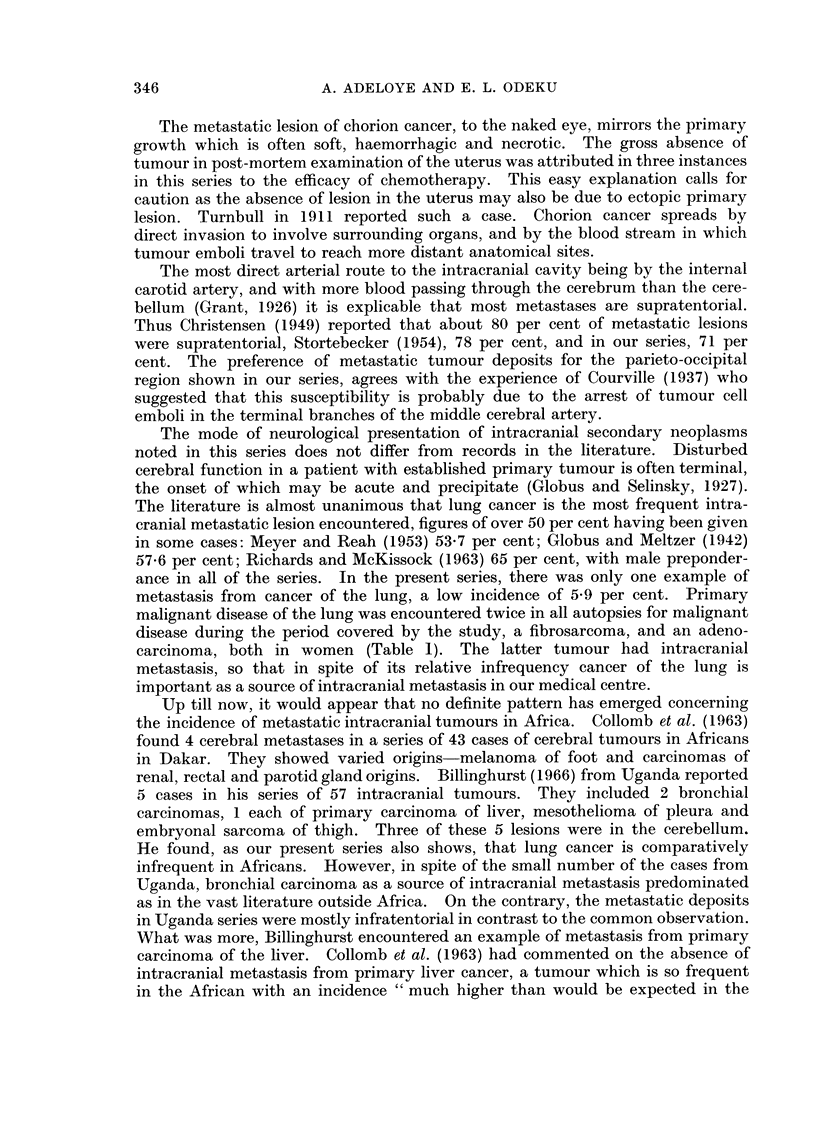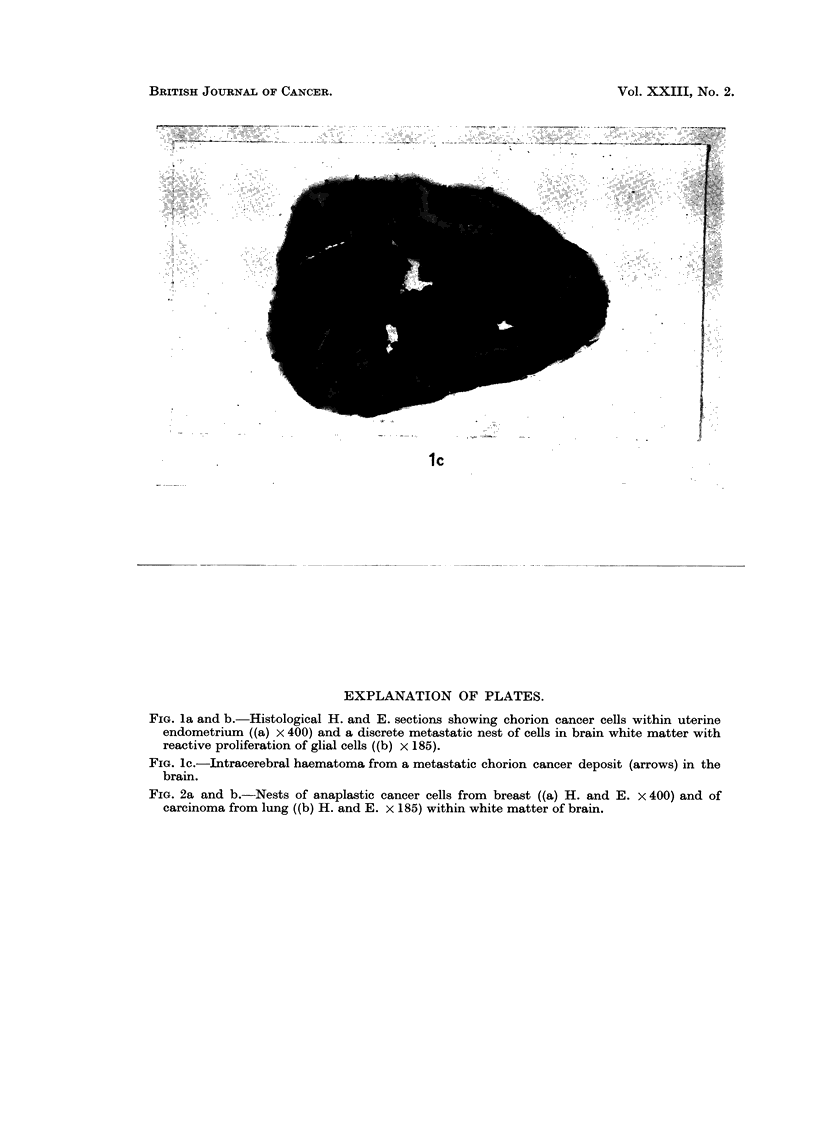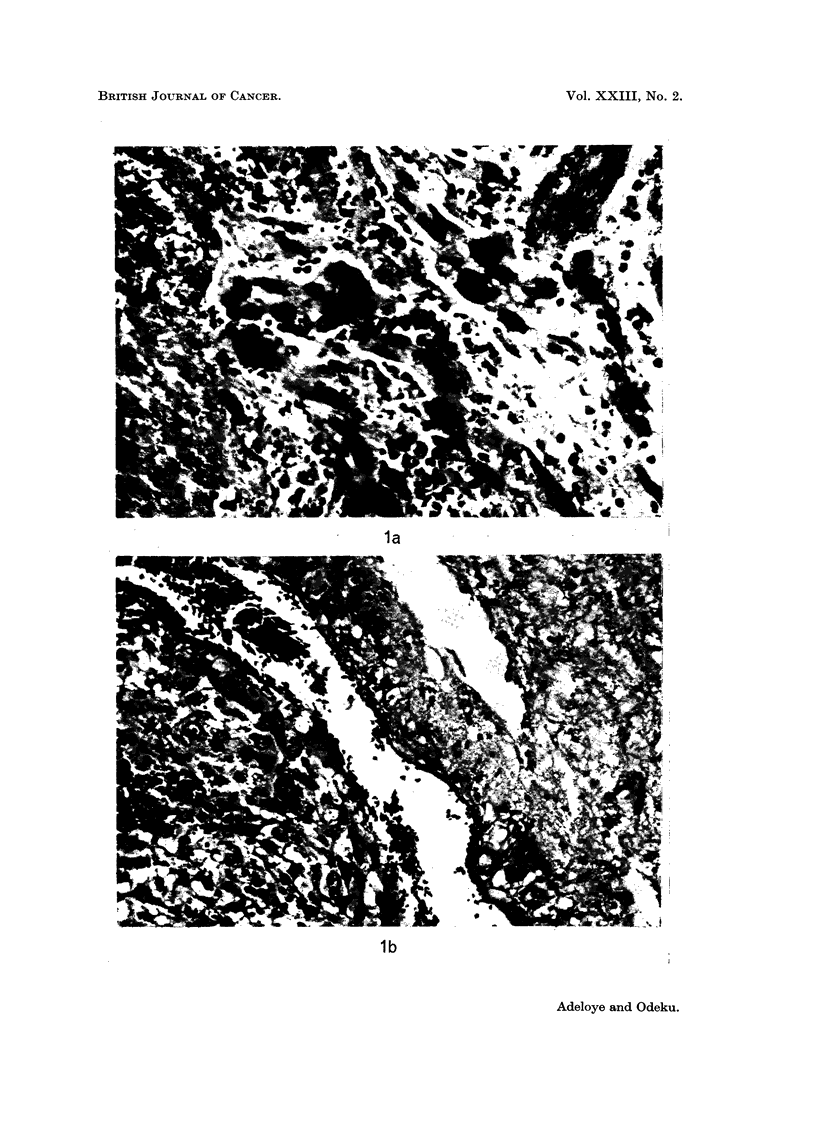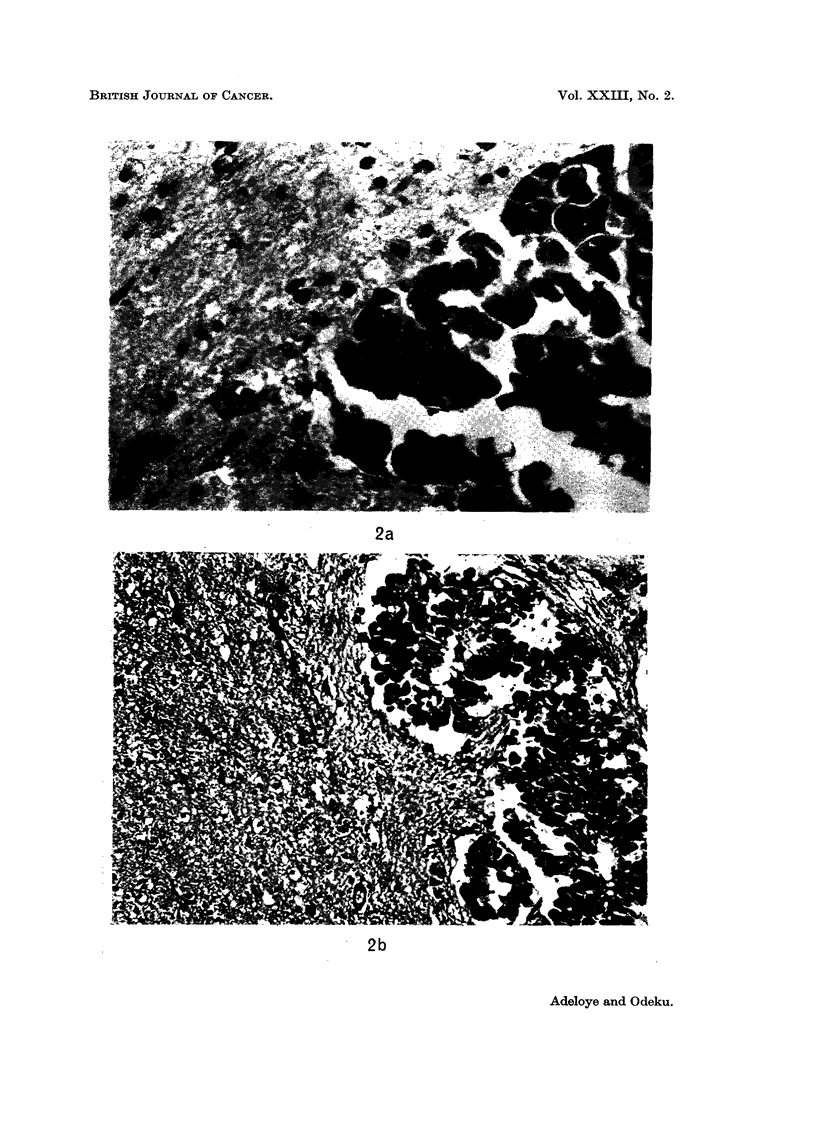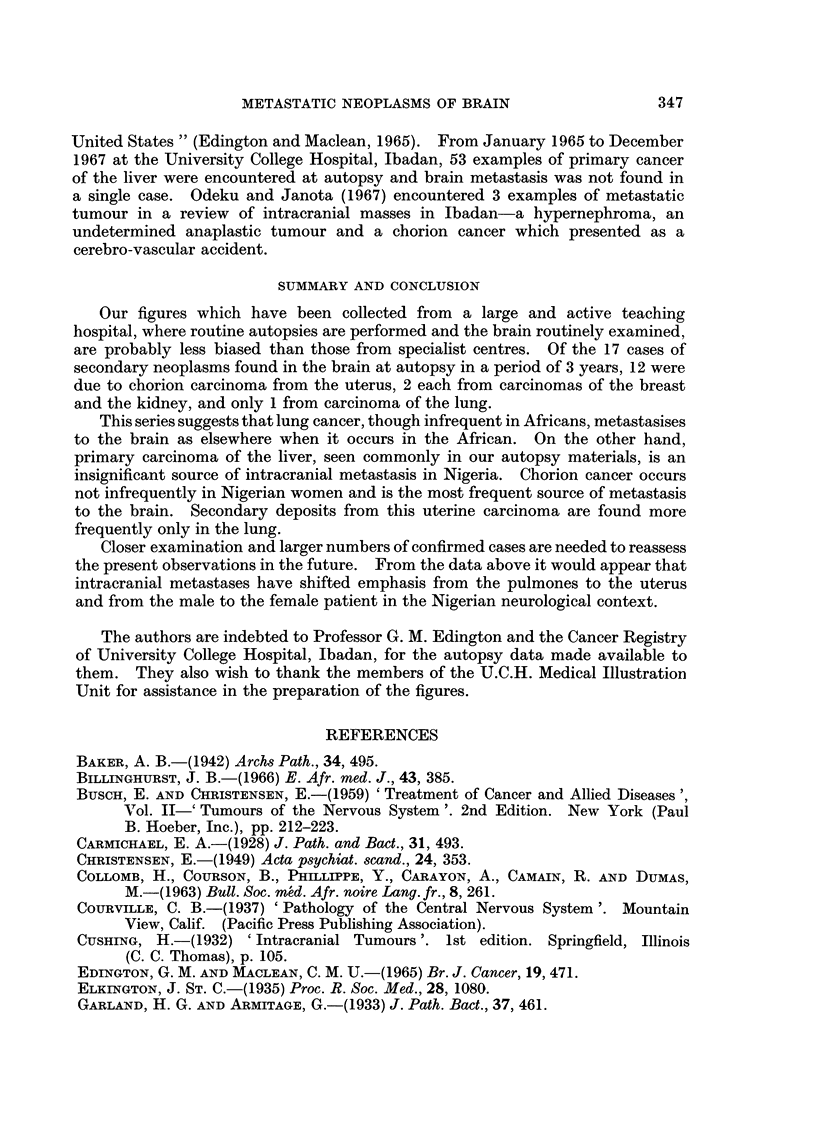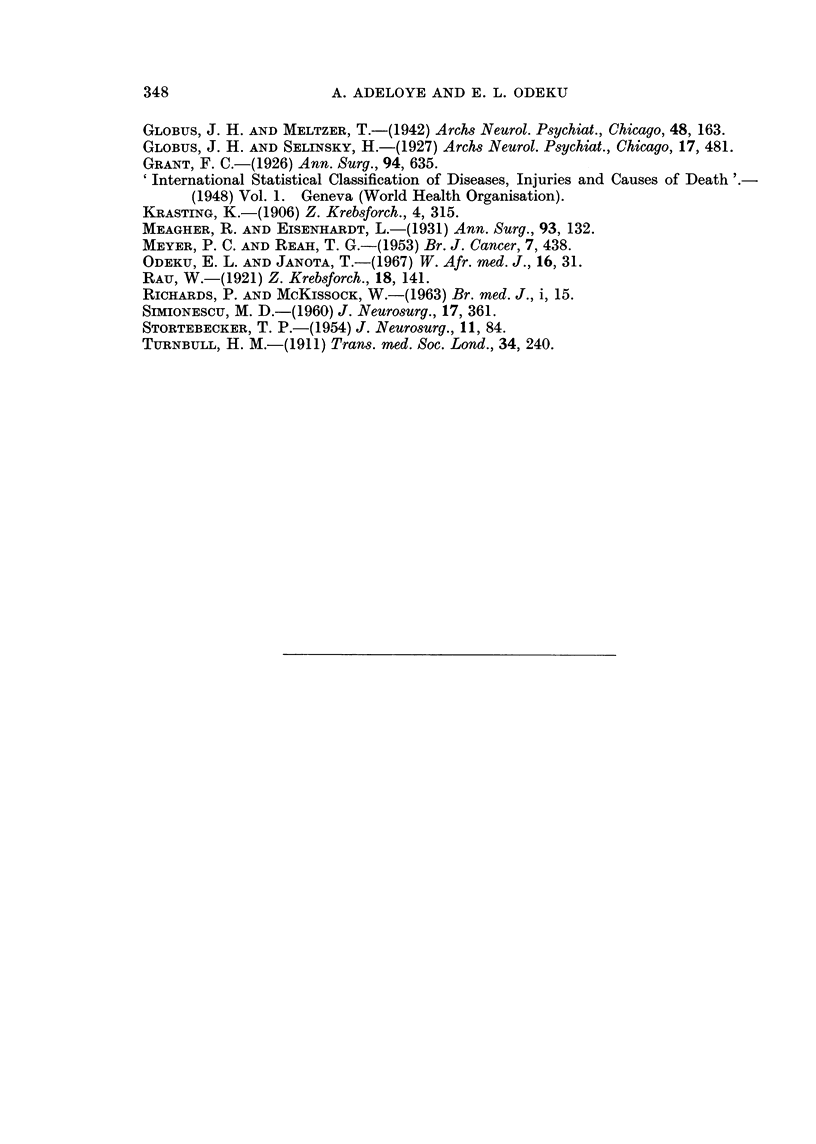# Metastatic neoplasms of the brain in Nigeria.

**DOI:** 10.1038/bjc.1969.44

**Published:** 1969-06

**Authors:** A. Adeloye, E. L. Odeku

## Abstract

**Images:**


### METASTATIC NEOPLASMS OF THE BRAIN IN NIGERIA

A. ADELOYE AND E. L. ODEKU

*From the Neurosurgery Unit, Surgery Department, University of Ibadan, Nigeria*

Received for publication February 24, 1969

SECONDARY neoplastic deposits in the brain have excited neurological interest since the first case was reported by Schraut in 1853 (Simionescu, 1960). The reported frequency in the literature has been very variable, depending on whether the material has been compiled from the autopsy room or a neurosurgical clinic. At the turn of the century, Krasting (1906) examined the brain in his series of 935 verified cases of cancer and found that there were 53 (5·6 per cent) instances of metastases. Rau (1921) reported 28 (3·2 per cent) intracranial metastases in a series of 851 autopsies in cases of cancer.

The relation of brain metastases to the total number in series of intracranial neoplasms was reported by Carmichael (1928) as 9·3 per cent. Garland and Armitage (1933) in Leeds recorded 12·8 per cent from 264 brain tumour autopsies; and Elkington (1935) at the National Hospital, Queen Square, London, found that metastases accounted for 72 (9 per cent) of 805 histologically proven brain tumours. Baker (1942) found more metastases (17·9 per cent) in his series of 114 cases of intracranial neoplasms at the University of Minnesota. Only 202 (6·3 per cent) of 3205 known brain tumours collected by Busch and Christensen (1959) were secondary. Reports from other neurosurgical clinics include 3 per cent from Meagher and Eisenhardt (1931), 3·2 per cent from Cushing (1932), 3·5 per cent from Stortebecker (1954), 4 per cent from Grant (1926) and 6·7 per cent from Simionescu (1960).

At neurosurgical clinics, the low frequency of metastatic tumours reflects the caution exercised by many surgeons who " refrain when possible from accepting patients with obvious intracranial metastasis ", (Cushing, 1932), and the feeling among others that " surgery, whether radical or palliative, is of no ultimate benefit to these patients in so far as prolongation of life is concerned " (Grant, 1926).

In Africa, where it has been suggested that intracranial neoplasms are rare, reports have been mostly of few isolated cases of primary tumours. Some short comprehensive series have yielded light preliminary data on the incidence of metastatic neoplasms of the brain in African indigenes. Collomb *et al.* (1963) in a study of 43 cases from the neuro-psychiatric service in Dakar noted that 5 or 11·6 per cent were metastases. Billinghurst (1966) in Kampala reported an incidence of 8·8 per cent; and more recently Odeku and Janota (1967) had an incidence of 6·5 per cent out of 46 intracranial masses from Nigeria.

The present communication places on record the incidence of metastases of the brain found at autopsy in the University College Hospital (U.C.H.), Ibadan, from January 1965 to December 1967.

### MATERIAL

During the period covered by this survey, autopsy was performed on 269 Nigerians with malignant neoplasms, all of which were histologically substantiated. The brain removed at autopsy in each case was suspended in 10 per cent formaldehyde solution (formalin). After an adequate fixation period usually lasting 3 to 4 weeks, the brain was carefully examined and sectioned in the coronal plane. Routine histological sections stained with haematoxylin and eosin were prepared from the brain and other relevant organs. Apart from the limitation in time-period, the material has been unselected.

### RESULTS

### *Relative incidence of the primary malignant tumours*

The 269 primary malignant tumours encountered at autopsy were classified according to the International Classification of Diseases (1948) (Table I). If

TABLE I.—*Distribution of the Primary Malignant Tumours Found at Autopsy in U.C.H. Ibadan January* 1965 *to December* 1967

| International classification | | | | |
|---|---|---|---|---|
| No. | Site of neoplasm | Males | Females | Total number |
| 145 | Oropharynx | — | 1 | 1 |
| 146 | Nasopharynx | 2 | — | 2 |
| 150 | Oesophagus | — | 1 | 1 |
| 151 | Stomach | 13 | — | 13 |
| 152 | Duodenum, Small intestine | 2 | 3 | 5 |
| 153 | Colon | 3 | 1 | 4 |
| 154 | Rectum | 1 | — | 1 |
| 155 | Primary liver | 44 | 9 | 53 |
| 155·1 | Gall bladder | — | 1 | 1 |
| 157 | Pancreas | 7 | — | 7 |
| 160 | Nasal sinuses | 1 | — | 1 |
| 161 | Larynx | 2 | — | 2 |
| 163 | Lung | — | 2 | 2 |
| 170 | Breast | — | 6 | 6 |
| 171 | Cervix uteri | — | 8 | 8 |
| 172 | Corpus uteri | — | 1 | 1 |
| 173·2 | Chorionepithelioma | — | 29 | 29 |
| 173 | Sarcoma of uterus | — | 1 | 1 |
| 175 | Carcinoma of ovary | — | 4 | 4 |
| 176 | Vulva and vagina | — | 2 | 2 |
| 177 | Prostate | 5 | — | 5 |
| 178 | Testis | 1 | — | 1 |
| 180 | Kidney | 4 | 3 | 7 |
| 181 | Bladder | 3 | 2 | 5 |
| 190 | Malignant melanoma | 1 | — | 1 |
| 192 | Retinoblastoma | — | 1 | 1 |
| 193 | Brain | 2 | 2 | 4 |
| 194 | Thyroid | — | 2 | 2 |
| 195 | Neuroblastoma | 1 | 1 | 2 |
| 197 | Kaposi's sarcoma | 1 | — | 1 |
| 199 | Unspecified | 1 | — | 1 |
| 200 | Reticulum cell sarcoma | — | 6 | 6 |
| 200·1 | Burkitt tumour | 12 | 10 | 22 |
| | Adult lymphosarcoma | 14 | 11 | 25 |
| 201 | Hodgkin's disease | 16 | 7 | 23 |
| 203 | Leukaemia | 12 | 5 | 17 |
| 204 | Multiple myeloma | 1 | 1 | 2 |
| | Total | 149 | 120 | 269 |

Burkitt tumour is grouped with those of the reticuloendothelial system, it will be seen from Table I that the commonest tumours encountered, in order of frequency, were reticuloendothelial tumours (34 per cent), primary cancer of the liver (19·7 per cent), and chorion carcinoma of the uterus (10·8 per cent). This pattern of incidence is broadly similar to the findings of Edington and Maclean (1965) during their cancer survey in Ibadan, Nigeria. Other notable primary tumours encountered were those of the kidneys (2·6 per cent), breast in females (2·2 per cent), brain (1·5 per cent) and lungs (0·7 per cent).

### *Metastatic tumour deposits in the brain*

There were 17 cases of metastatic tumour deposits in the brain in this series. Excluding the 4 cases of primary neoplasm of the brain from the 269 cases, this represents an incidence of 6·4 per cent. The clinico-pathological features of these 17 cases are considered below.

#### *Age and sex incidence*

The youngest example was a 7 year old girl with Wilms' tumour of the right kidney with cerebellar metastasis, and the oldest, a woman of 60 with adeno-carcinoma of the right lung with a metastasis in the right cerebral hemisphere. Fourteen cases (82·4 per cent) were in the 3rd and 4th decades of life and 1 case each in the 1st, 5th and 6th decades of life (Table II). All of our 17 cases were females.

TABLE II.—*Age Incidence of Secondary Neoplasms in Brain*

| Age in years | Number of cases |
|---|---|
| 0–10 | 1 |
| 11–20 | — |
| 21–30 | 5 |
| 31–40 | 9 |
| 41–50 | 1 |
| 51–60 | 1 |
| Total | 17 |

#### *Sites of primary growth giving rise to brain metastases*

The primary sites of these neoplasms are listed in order of frequency in Table III. Metastases from chorion cancer of the uterus were by far the commonest

TABLE III.—*Sites of Primary Growth*

| Primary site | Histological type | No. of cases | Per cent incidence |
|---|---|---|---|
| Uterus | Chorion cancer | 12 | 70·5 |
| Breast | Adenocarcinoma | 2 | 11·8 |
| Kidney | (a) Hypernephroma<br>(b) Wilms' tumour | 2 | 11·8 |
| Lung | Adenocarcinoma | 1 | 5·9 |

(70·5 per cent) in this series. Metastases from breast and kidney neoplasms were each encountered in 11·8 per cent of cases. There was only one case of metastasis from adenocarcinoma of the lung.

*Intracranial localisation of metastases*

The lesions were solitary in 11 cases (64 per cent) and multiple in the rest. In 12 cases (70·5 per cent) the metastases were supratentorial. In half of the cases in which metastases were multiple, there were cerebellar deposits in association with the supratentorial lesions. Of the supratentorial lesions, 57 per cent occurred in the left cerebral hemisphere. The specific involvement of the various structures bearing the secondary lesions within the brain is summarised in Table IV.

TABLE IV.—*Location of Metastatic Lesions in the Brain*

| Location | No. of cases |
|---|---|
| Parietal region | 7 |
| Occipital region | 5 |
| Frontal region | 4 |
| Cerebellum | 3 |
| Basal ganglia | 2 |
| Temporal region | 1 |
| Pituitary | 1 |

*Clinico-pathological aspects*

I. *Chorion carcinoma group*

The 12 cases which comprised this group were in the 3rd and 4th decades. Death seemed to follow the onset of intracranial symptoms and signs rapidly, the time interval between onset of these clinical features and the demise of the patient varying from 6 days to 4 weeks.

The development of psychosis was often the inaugural pointer to intracranial involvement in the patient with established chorion cancer. Headache, although encountered more often, usually came after the onset of the mental confusion. The site of the headache often corresponded to that of the metastases. Next in the sequence of events was the occurrence of epileptic seizures, usually unilateral Jacksonian seizures. In the five instances in which Jacksonian fits were recorded, they occurred 10 days, 10 days, 5 days, 2 days, and 12 hours before death, almost contemporaneously as profound alteration in the level of consciousness of the patient was noted. The above sequence of clinical events was fairly well exemplified by the case described immediately below.

CASE NO. 1

This 33 year old woman aborted a 2 month old pregnancy and bled *per vaginam* for 30 days. A month later she developed an unproductive cough and felt pain in the left side of her chest. Chorion cancer was diagnosed and confirmed histologically. Clinically there was no neurological deficit. She was treated with methotrexate and 6-mercaptopurine. A month before death she became mentally confused, answered questions incoherently and threatened to jump out of bed. The change in mood was first attributed to the fever which she developed after the chemotherapy. A week later there was a Jacksonian fit which involved the right half of the face. She became stuporose and died in coma a week thereafter.

*Autopsy.*—The middle lobe of the right lung, which weighed 656 g., showed a cystic haemorrhagic lesion measuring 2 × 1 × 1 cm. The left lung weighed 1240 g. and its cut surface also presented a circumscribed tumour mass involving the entire lower lobe. It measured 15 × 10 × 8 cm. and on its pink cut surface, there were areas of haemorrhage and necrosis. The uterine endometrium was congested but serial sections of the uterus revealed no tumour. The brain weighed 1365 g., and in its left hemisphere there was a discrete haemorrhagic lesion measuring 4 × 3 × 2 cm. Histological sections of part of the uterus suspected to have neoplastic tissue and of the lesion from the brain were identical (Fig. 1a and b). The absence of tumour tissue in the uterus on serial sectioning at autopsy was encountered in 3 cases, all of which had courses of chemotherapy. Antemortem histological examination, however, demonstrated the uterine lesion before chemotherapy was instituted.

In 4 cases with supratentorial metastases, there were no symptoms referable to the central nervous system. Three of the lesions were solitary and discrete and the fourth was multiple. An interesting mode of presentation of intracranial metastatic chorion cancer is the picture of acute cerebrovascular accident (haemorrhage). Two patients presented in a stroke fashion in this group and one of these is described below.

CASE NO. 2

A 40 year old housewife was admitted in coma of about 12 hours duration. Her past medical history was unremarkable. On examination, the head was turned to the left and the neck was rigid. She also had right facial weakness of the upper motor neurone type and the right limbs were paralysed. The blood pressure was 110/70 mm. Hg and hepatosplenomegaly (2 fingers breadth each) was noted. Right carotid angiography showed a shift of the anterior cerebral vessels to the right. Some abnormal angiomatous-looking vessels were seen in the left parietal region. What was grossly regarded as an intracerebral haematoma, comprising dark liquid and organised blood, was removed through a left fronto-parietal craniotomy. No neoplasm was suspected. Patient died 24 hours after operation.

*Autopsy.*—The uterus was found to be enlarged (size of one month pregnancy) and the uterine cavity was distended by a haemorrhagic polypoid mass which arose from the uterine fundus and was about 3·5 cm. in diameter. A firm nodule of pale tissue, about 4 cm. in diameter and surrounded by haemorrhage and necrosis, was present in the lower lobe of the right lung. The brain contained a left parietal haemorrhagic mass which was histologically shown to be chorion carcinoma.

During the period covered by the present study, autopsy was performed on 29 individuals with chorion carcinoma. The organs invaded by the metastases from this cancer are shown in Table V, the lung being the most frequently involved (82·7 per cent). Metastases to the brain ranked next at 41·4 per cent. Fig. 1c shows the gross appearance of chorion cancer deposit and secondary haemorrhage from it into a brain which was examined recently.

II. *Other groups*

*Metastasis from carcinoma of the breast.*—There were two examples in this series, both in pre-menopausal women. One was a stage IV poorly differentiated squamous cell carcinoma of the left breast. She had simple mastectomy for her fungating growth and bilateral oophorectomy was performed subsequently.

A month before death she developed right facial weakness of an upper motor neurone type and right hemiplegia. A week later blatant personality changes were noted. Autopsy showed metastatic deposits in lungs, liver, pituitary gland, and the brain (Fig. 2a).

*Metastasis from tumours of the kidney.*—There were two instances, one of which was a hypernephroma arising from the left kidney in a woman of 40, with metastatic lesions in both lungs and in the frontal lobe of the brain. The other example was a female of 7 with a massive haemorrhagic Wilms' tumour of the right kidney, spreading to the liver, left kidney and the left cerebellum. The cerebellar mass was 2 × 1·5 × 1·5 cm. and tonsillar pressure cones were prominent.

*Metastasis from carcinoma of the lung.*—There was only one example, a primary adenocarcinoma of the right lung with secondary deposits in the liver, right kidney and the brain (Fig. 2b). In the brain there were circumscribed nodules of tumour tissue in the cortex and white matter of the right parietal lobe and also in the right middle cerebellar peduncle.

### DISCUSSION

The significant note in this unselected group of neoplasms is the overwhelming preponderance of intracranial metastases from chorion cancer, with an incidence of 70·5 per cent. Nowhere in the literature has such a high incidence been recorded. Meyer and Reah (1953) in 216 autopsy cases of secondary neoplasm of the central nervous system and meninges, reported only 6 cases of metastases from chorion cancer, a percentage incidence of 2·8.

An extremely malignant growth of trophoblastic origin, chorion cancer has been found to be notorious for its widespread dissemination. During the period covered by the present study, 25 (86 per cent) of the 29 patients with chorion cancer who came to autopsy had metastases. In the spread of chorion cancer no organ seems exempt as shown by Table V. The lung was most frequently

TABLE V.—*Organs with Metastatic Chorion Carcinoma (Autopsies on 29 Cases)*

| Organ | No. of cases |
|---|---|
| Lungs | 24 |
| Brain | 12 |
| Liver | 7 |
| Kidney | 6 |
| Vagina | 3 |
| Lymph nodes | 3 |
| Peritoneum | 2 |
| Intestine | 2 |
| Bladder | 1 |
| Pancreas | 1 |
| Urethra | 1 |
| Cervix | 1 |
| Spleen | 1 |
| Stomach | 1 |
| Heart | 1 |

involved (82·7 per cent), next the brain which was involved in 12 cases (41·4 per cent) out of the total 29. Hendrickse (1968, personal communication) in a review of fatal cases of chorion cancer at the University College Hospital, Ibadan, from January 1957 to December 1965, found the brain involved in 9 out of 34 cases, an incidence of 27 per cent.

The metastatic lesion of chorion cancer, to the naked eye, mirrors the primary growth which is often soft, haemorrhagic and necrotic. The gross absence of tumour in post-mortem examination of the uterus was attributed in three instances in this series to the efficacy of chemotherapy. This easy explanation calls for caution as the absence of lesion in the uterus may also be due to ectopic primary lesion. Turnbull in 1911 reported such a case. Chorion cancer spreads by direct invasion to involve surrounding organs, and by the blood stream in which tumour emboli travel to reach more distant anatomical sites.

The most direct arterial route to the intracranial cavity being by the internal carotid artery, and with more blood passing through the cerebrum than the cerebellum (Grant, 1926) it is explicable that most metastases are supratentorial. Thus Christensen (1949) reported that about 80 per cent of metastatic lesions were supratentorial, Stortebecker (1954), 78 per cent, and in our series, 71 per cent. The preference of metastatic tumour deposits for the parieto-occipital region shown in our series, agrees with the experience of Courville (1937) who suggested that this susceptibility is probably due to the arrest of tumour cell emboli in the terminal branches of the middle cerebral artery.

The mode of neurological presentation of intracranial secondary neoplasms noted in this series does not differ from records in the literature. Disturbed cerebral function in a patient with established primary tumour is often terminal, the onset of which may be acute and precipitate (Globus and Selinsky, 1927). The literature is almost unanimous that lung cancer is the most frequent intracranial metastatic lesion encountered, figures of over 50 per cent having been given in some cases: Meyer and Reah (1953) 53·7 per cent; Globus and Meltzer (1942) 57·6 per cent; Richards and McKissock (1963) 65 per cent, with male preponderance in all of the series. In the present series, there was only one example of metastasis from cancer of the lung, a low incidence of 5·9 per cent. Primary malignant disease of the lung was encountered twice in all autopsies for malignant disease during the period covered by the study, a fibrosarcoma, and an adenocarcinoma, both in women (Table 1). The latter tumour had intracranial metastasis, so that in spite of its relative infrequency cancer of the lung is important as a source of intracranial metastasis in our medical centre.

Up till now, it would appear that no definite pattern has emerged concerning the incidence of metastatic intracranial tumours in Africa. Collomb *et al.* (1963) found 4 cerebral metastases in a series of 43 cases of cerebral tumours in Africans in Dakar. They showed varied origins—melanoma of foot and carcinomas of renal, rectal and parotid gland origins. Billinghurst (1966) from Uganda reported 5 cases in his series of 57 intracranial tumours. They included 2 bronchial carcinomas, 1 each of primary carcinoma of liver, mesothelioma of pleura and embryonal sarcoma of thigh. Three of these 5 lesions were in the cerebellum. He found, as our present series also shows, that lung cancer is comparatively infrequent in Africans. However, in spite of the small number of the cases from Uganda, bronchial carcinoma as a source of intracranial metastasis predominated as in the vast literature outside Africa. On the contrary, the metastatic deposits in Uganda series were mostly infratentorial in contrast to the common observation. What was more, Billinghurst encountered an example of metastasis from primary carcinoma of the liver. Collomb *et al.* (1963) had commented on the absence of intracranial metastasis from primary liver cancer, a tumour which is so frequent in the African with an incidence " much higher than would be expected in the

1c

### EXPLANATION OF PLATES.

FIG. 1a and b.—Histological H. and E. sections showing chorion cancer cells within uterine endometrium ((a) × 400) and a discrete metastatic nest of cells in brain white matter with reactive proliferation of glial cells ((b) × 185).

FIG. 1c.—Intracerebral haematoma from a metastatic chorion cancer deposit (arrows) in the brain.

FIG. 2a and b.—Nests of anaplastic cancer cells from breast ((a) H. and E. × 400) and of carcinoma from lung ((b) H. and E. × 185) within white matter of brain.

1a

1b

2a

2b

Adeloye and Odeku.

United States " (Edington and Maclean, 1965). From January 1965 to December 1967 at the University College Hospital, Ibadan, 53 examples of primary cancer of the liver were encountered at autopsy and brain metastasis was not found in a single case. Odeku and Janota (1967) encountered 3 examples of metastatic tumour in a review of intracranial masses in Ibadan—a hypernephroma, an undetermined anaplastic tumour and a chorion cancer which presented as a cerebro-vascular accident.

### SUMMARY AND CONCLUSION

Our figures which have been collected from a large and active teaching hospital, where routine autopsies are performed and the brain routinely examined, are probably less biased than those from specialist centres. Of the 17 cases of secondary neoplasms found in the brain at autopsy in a period of 3 years, 12 were due to chorion carcinoma from the uterus, 2 each from carcinomas of the breast and the kidney, and only 1 from carcinoma of the lung.

This series suggests that lung cancer, though infrequent in Africans, metastasises to the brain as elsewhere when it occurs in the African. On the other hand, primary carcinoma of the liver, seen commonly in our autopsy materials, is an insignificant source of intracranial metastasis in Nigeria. Chorion cancer occurs not infrequently in Nigerian women and is the most frequent source of metastasis to the brain. Secondary deposits from this uterine carcinoma are found more frequently only in the lung.

Closer examination and larger numbers of confirmed cases are needed to reassess the present observations in the future. From the data above it would appear that intracranial metastases have shifted emphasis from the pulmones to the uterus and from the male to the female patient in the Nigerian neurological context.

The authors are indebted to Professor G. M. Edington and the Cancer Registry of University College Hospital, Ibadan, for the autopsy data made available to them. They also wish to thank the members of the U.C.H. Medical Illustration Unit for assistance in the preparation of the figures.

### REFERENCES

Baker, A. B.—(1942) *Archs Path.*, **34**, 495.

Billinghurst, J. B.—(1966) *E. Afr. med. J.*, **43**, 385.

Busch, E. and Christensen, E.—(1959) 'Treatment of Cancer and Allied Diseases', Vol. II—'Tumours of the Nervous System'. 2nd Edition. New York (Paul B. Hoeber, Inc.), pp. 212–223.

Carmichael, E. A.—(1928) *J. Path. and Bact.*, **31**, 493.

Christensen, E.—(1949) *Acta psychiat. scand.*, **24**, 353.

Collomb, H., Courson, B., Phillippe, Y., Carayon, A., Camain, R. and Dumas, M.—(1963) *Bull. Soc. méd. Afr. noire Lang. fr.*, **8**, 261.

Courville, C. B.—(1937) 'Pathology of the Central Nervous System'. Mountain View, Calif. (Pacific Press Publishing Association).

Cushing, H.—(1932) 'Intracranial Tumours'. 1st edition. Springfield, Illinois (C. C. Thomas), p. 105.

Edington, G. M. and Maclean, C. M. U.—(1965) *Br. J. Cancer*, **19**, 471.

Elkington, J. St. C.—(1935) *Proc. R. Soc. Med.*, **28**, 1080.

Garland, H. G. and Armitage, G.—(1933) *J. Path. Bact.*, **37**, 461.

GLOBUS, J. H. AND MELTZER, T.—(1942) *Archs Neurol. Psychiat., Chicago*, **48**, 163.
GLOBUS, J. H. AND SELINSKY, H.—(1927) *Archs Neurol. Psychiat., Chicago*, **17**, 481.
GRANT, F. C.—(1926) *Ann. Surg.*, **94**, 635.
'International Statistical Classification of Diseases, Injuries and Causes of Death'.—(1948) Vol. 1. Geneva (World Health Organisation).
KRASTING, K.—(1906) *Z. Krebsforch.*, 4, 315.
MEAGHER, R. AND EISENHARDT, L.—(1931) *Ann. Surg.*, **93**, 132.
MEYER, P. C. AND REAH, T. G.—(1953) *Br. J. Cancer*, **7**, 438.
ODEKU, E. L. AND JANOTA, T.—(1967) *W. Afr. med. J.*, **16**, 31.
RAU, W.—(1921) *Z. Krebsforch.*, **18**, 141.
RICHARDS, P. AND MCKISSOCK, W.—(1963) *Br. med. J.*, i, 15.
SIMIONESCU, M. D.—(1960) *J. Neurosurg.*, **17**, 361.
STORTEBECKER, T. P.—(1954) *J. Neurosurg.*, **11**, 84.
TURNBULL, H. M.—(1911) *Trans. med. Soc. Lond.*, **34**, 240.